# Extended dosing (12 cycles) vs conventional dosing (6 cycles) of adjuvant temozolomide in adults with newly diagnosed high-grade gliomas: a randomized, single-blind, two-arm, parallel-group controlled trial

**DOI:** 10.3389/fonc.2024.1357789

**Published:** 2024-05-07

**Authors:** Kazem Anvari, Mehdi Seilanian Toussi, Mohammadreza Saghafi, Seyed Alireza Javadinia, Hamidreza Saghafi, James S. Welsh

**Affiliations:** ^1^ Cancer Research Center, Faculty of Medicine, Mashhad University of Medical Sciences, Mashhad, Iran; ^2^ Department of Urology, Roswell Park Comprehensive Cancer Center, Buffalo, NY, United States; ^3^ Qazvin University of Medical Sciences, Qazvin, Qazvin, Iran; ^4^ Non-Communicable Diseases Research Center, Sabzevar University of Medical Sciences, Sabzevar, Iran; ^5^ Faculty of Medicine, Tehran Medical Branch of Islamic Azad University, Tehran, Iran; ^6^ Department of Radiation Oncology, Loyola University Chicago Stritch School of Medicine, Edward Hines Jr., VA Hospital, Maywood, IL, United States

**Keywords:** high grade gliomas, adjuvant temozolomide, survival, extended chemotherapy, randomized controlled trial, glioblastoma multiforme

## Abstract

**Purpose:**

Maximum safe surgical resection followed by adjuvant chemoradiation and temozolomide chemotherapy is the current standard of care in the management of newly diagnosed high grade glioma. However, there are controversies about the optimal number of adjuvant temozolomide cycles. This study aimed to compare the survival benefits of 12 cycles against 6 cycles of adjuvant temozolomide adults with newly diagnosed high grade gliomas.

**Methods:**

Adult patients with newly diagnosed high grade gliomas, and a Karnofsky performance status>60%, were randomized to receive either 6 cycles or 12 cycles of adjuvant temozolomide. Patients were followed-up for assessment of overall survival (OS) and progression-free survival (PFS) by brain MRI every 3 months within the first year after treatment and then every six months.

**Results:**

A total of 100 patients (6 cycles, 50; 12 cycles, 50) were entered. The rate of treatment completion in 6 cycles and 12 cycles groups were 91.3% and 55.1%, respectively. With a median follow-up of 26 months, the 12-, 24-, 36-, and 48-month OS rates in 6 cycles and 12 cycles groups were 81.3% vs 78.8%, 58.3% vs 49.8%, 47.6% vs 34.1%, and 47.6% vs 31.5%, respectively (p-value=.19). Median OS of 6 cycles and 12 cycles groups were 35 months (95% confidence interval (CI), 11.0 to 58.9) and 23 months (95%CI, 16.9 to 29.0). The 12-, 24-, 36-, and 48- month PFS rates in 6 cycles and 12 cycles groups were 70.8% vs 56.9%, 39.5% and 32.7%, 27.1% vs 28.8%, and 21.1% vs 28.8%, respectively (p=.88). The Median PFS of 6 cycles and 12 cycles groups was 18 months (95% CI, 14.8 to 21.1) and 16 (95% CI, 11.0 to 20.9) months.

**Conclusion:**

Patients with newly diagnosed high grade gliomas treated with adjuvant temozolomide after maximum safe surgical resection and adjuvant chemoradiation do not benefit from extended adjuvant temozolomide beyond 6 cycles.

**Trial registration:**

Prospectively registered with the Iranian Registry of Clinical Trials: IRCT20160706028815N3. Date registered: 18/03/14.

## 1 Introduction

Glioblastoma is the most common and most aggressive tumor primary brain tumor originating from astrocytes. With a 3-year survival of approximately 10 percent, the dire prognosis of this tumor has not improved significantly in recent years (1). Complete resection of this tumor is almost impossible due to the invasive nature of the tumor and involvement of eloquent parts of the brain. Adjuvant post-surgical treatment is required to prevent or delay recurrence.

The current standard of care for newly diagnosed patients with appropriate performance status is maximum safe surgical resection, post-operative radiation concomitant with daily oral temozolomide and adjuvant chemotherapy with temozolomide (2). This recommendation is based on a randomized trial that recorded a significant benefit of the combined temozolomide and radiotherapy protocol over the radiotherapy alone arm (overall survival at 2 years, 27.2% versus 10.9%) (3). In this standard protocol (sometimes called the Stupp regimen), 3-dimentional radiotherapy of 60 Gy in 30 fractions is administered concomitant with temozolomide (75 mg/m^2^, 7 days per week). Then, after 4 weeks from radiation termination, patients receive up to six courses of adjuvant temozolomide (150-200 mg/m^2^ for 5 days every 28 days). A cohort retrospective cohort study using linked population bases from a cancer registry in Australia, revealed that patients treated in 2010-2012 (which corresponds to the era of temozolomide use), had better median survival than those treated in the pre-temozolomide period of 2001-2003 (10.6 months vs. 7.4 months) (4). However, these figures confirm that the prognosis of patients with glioblastoma has remained dismal. To date, the trials on using other treatment modalities like stereotactic radiosurgery, heavy charged particles, interstitial brachytherapy, tumor treating fields (TT-Fields), antiangiogenic therapy, immunotherapy, and gene therapy have shown inconsistent or disappointing results (5). In an attempt to improve survival, adjuvant dose dense adjuvant temozolomide (75 mg/m^2^ on days 1 to 21 every 28 days) has been compared against standard adjuvant protocol with no encouraging result (6, 7).

Considering lack of effective salvage treatment in case of recurrence, it has been a usual practice in many centers to extend the adjuvant temozolomide beyond 6 courses up to 12, especially in patients who tolerate well the treatment well with no evidence of progression (8–11). The results of retrospective and randomized trials on the benefit of such extended adjuvant treatment have not been consistent. Extended treatment may increase the toxicity of treatment and it worsens the economic burden of treatment (12). Furthermore, there are reports that continued treatment may induce resistance to the ongoing alkylating agents and alters response to salvage therapy (13, 14).

In this prospective randomized trial, we aimed to assess the feasibility of extended adjuvant temozolomide (12 courses) and compare it against standard treatment (6 courses) in eligible patients with glioblastoma.

## 2 Methods

### 2.1 Participants

The study was conducted at three main tertiary referral Cancer Treatment Centers of Mashhad, Iran including the Oncology Clinics of Imam Reza and Omid Educational Hospitals both affiliated with Mashhad University of Medical Sciences as well as Reza Radiotherapy Oncology Center between April 2018 and October 2020.We enrolled newly diagnosed patients with pathologically confirmed glioblastoma multiforme or anaplastic astrocytoma who had undergone tumor resection, had a Karnofsky performance status of >60%, normal kidney and liver functions tests, and adequate bone marrow capacity. Patients were excluded in cases with a previous history of malignancy, previous treatment with chemotherapy and/or radiotherapy, and if they selected strictly palliative treatment (receiving radiation therapy alone or altered fractionated radiotherapy).

### 2.2 Study design

In this randomized, single-blind, parallel-group trial, we assigned the eligible patients at the commencement of chemoradiation to receive either 6 cycles or 12 cycles of adjuvant temozolomide by block randomization [2:2]. In this context, the letter A or letter B was allocated to the 6 cycles or 12 cycles groups, drawing four potential combinations (i.e., AABB, BBAA, ABAB, and BABA). The envelope randomization method was used to assign patients to each group.

Before starting the treatment, all patients underwent brain MRI to assess the residual tumor. We performed staging work-up according to the last version of the National Comprehensive Cancer Network (NCCN) guidelines for Central Nervous System Cancers (15, 16). Brain CT scan with contrast with a slice separation of 5 mm was obtained for 3D conformal radiotherapy planning. Treatment planning was performed using Radiation Therapy Oncology Group (RTOG) two phase or European Organization for Research and Treatment of Cancer (EORTC) single phase recommendations for target definition (1). All patients received focal external beam irradiation of 60 Gy in 30 fractions. The patients received concurrent temozolomide (75 mg/m^2^, daily) based on Stupp’s protocol (2). Adjuvant chemotherapy with single agent oral temozolomide (150-200 mg/m^2^, the first to fifth day, every 28 days) was started four weeks after the completion of chemoradiation.Adjuvant temozolomide was initiated at a dose of 150 mg/m^2^ and the doses was increased to 200 mg/m^2^ and continued at this dose if there was an absence of any grade 2-4 hematologic toxicities. The patients received ondansetron (4 mg every 8 hours) as antiemetic prophylaxis during the concurrent chemoradiation, and the adjuvant chemotherapy. Before each course of chemotherapy, patients were inquired about their signs and symptoms, underwent physical examination and Complete Blood Count (CBC) to assess the treatment toxicity, as well as signs and symptoms of the disease progression/recurrence. Moreover, a brain MRI with gadolinium contrast was obtained every 3 months within the first year after treatment termination, then every six months to detect possible local disease progression/recurrence.

### 2.3 Variables

#### 2.3.1 Survival analysis

The time interval in months between the first pathologic diagnosis and the first evidence of disease recurrence (presence of newly enhancive tumoral lesion within or outside of radiotherapy field) or disease progression (an increase in enhancive lesion size by 25 percent) was considered as the progression-free survival (PFS) (3). The overall survival (OS) was defined as the time interval in months between the initial pathologic diagnosis and death/last visit.

#### 2.3.2 Treatment toxicity

The Eastern Cooperative Oncology Group Common Toxicity Criteria V 5.0 (ECOG-CTC) was used to assess chemotherapy-induced neutropenia, thrombocytopenia, anemia, constipation, diarrhea, nausea, vomiting, and alopecia using a 4-grade scoring system (through mildest to most severe; grade 0 to grade 4).

### 2.4 Ethics

This trial was registered in the Iranian Registration of clinical trials (IRCT20160706028815N3), prospectively. The study protocol was approved by the Ethics Committee of Mashhad University of Medical Sciences (approval code: IR.MUMS.fm.REC.1396.449) and was conducted according to the Declaration of Helsinki. Undersigned informed consent forms were obtained from all patients prior to the enrollment.

### 2.5 Statistical analyses and sample size

#### 2.5.1 Sample size

Considering the 12-month survival rate of 82.9% and 100% in patients with high-grade glioma receiving 6 or more than 6- cycles of adjuvant temozolomide respectively in a previous study (4), with a type I error rate of.05 and statistical power of 80%, the sample size was calculated to be 31 patients in each group ( $n=\frac{{\left(Z_{1-\frac{\alpha }{2\;}}+Z_{1-\beta }\right)}^{2}\left(P_{1}\left(1-P_{1}\right)+P_{2}\left(1-P_{2}\right)\right)}{{\left(d\right)}^{2}}$ ). However, due to potential loss to follow-up, we designed the trial to enroll at least 50 patients in each group.

#### 2.5.2 Statistical analyses

The normality of data was assessed by Shapiro–Wilk test using the Statistical Package for Social Science version 22 (SPSS Inc., Chicago, Illinois). All data had a normal distribution. Therefore, categorical data and quantitative data were analyzed using Chi square (Fisher’s exact test) and t test, respectively. Intention-to-treat analysis was adopted to perform statistical analysis. The survival data were presented by Kaplan-Meier curves and were analyzed by univariate log-rank (5). A p-value <0.05 was considered statistically significant. Moreover, Multivariate Cox regression analysis was used to detect the contributing factors to overall survival of patients with high-grade glioma.

## 3 Results

### 3.1 Patients

**Figure 1:**
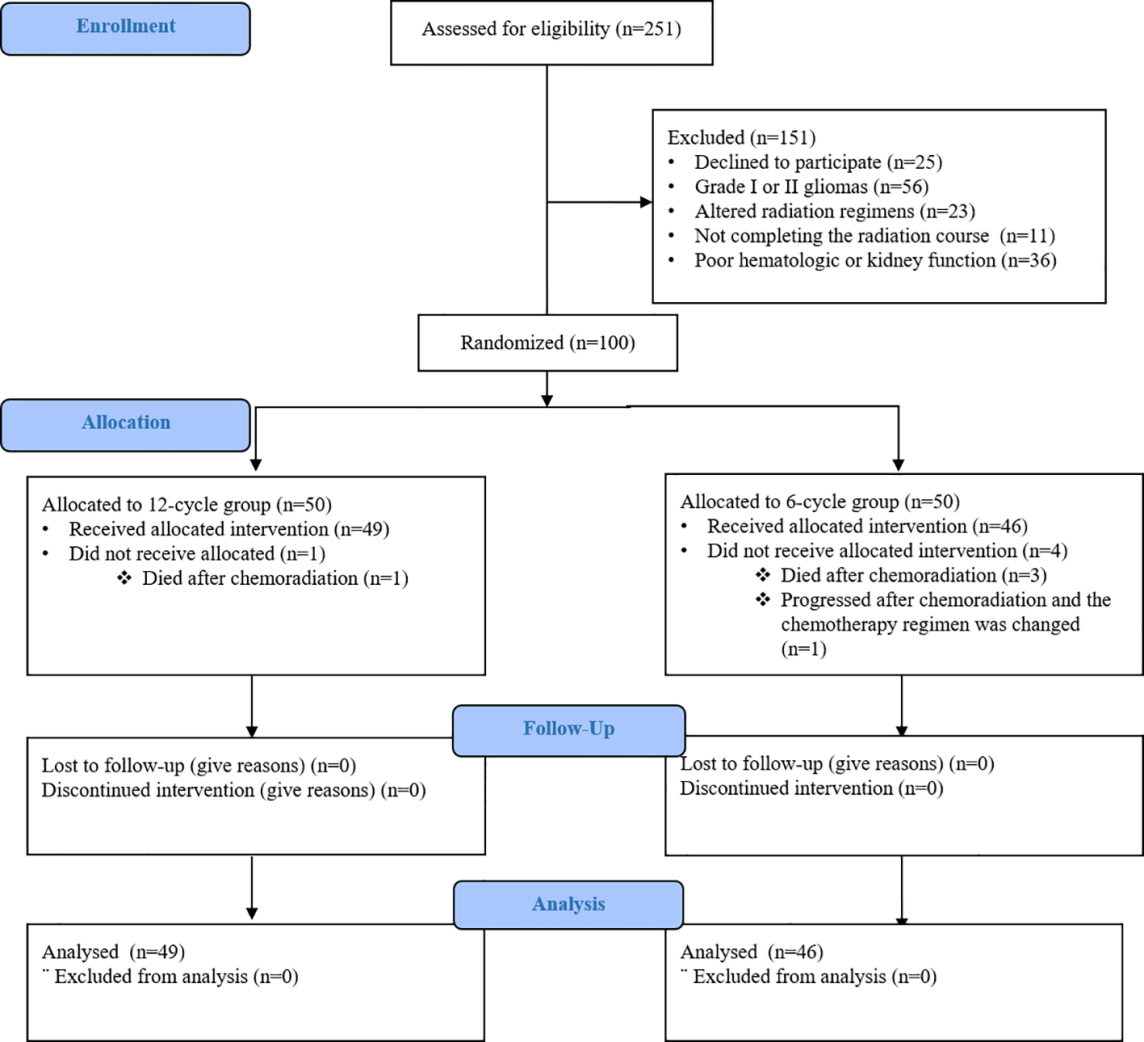
Consort flow diagram.

From April 2018 until October 2020, 100 patients from 3 institutions in Mashhad, Iran were randomly assigned to receive 6 cycles (50 patients) or 12 cycles of adjuvant temozolomide (50 patients). In 6-cycle group, three patients died after the completion of chemoradiation and the chemotherapy regimen of one patient was changed to bevacizumab-based chemotherapy due to disease progression before the first course of adjuvant temozolomide. In 12-cycle group, only one patient did not receive allocation since he died after the completion of chemoradiation (**Figure 1**).

Both groups were similar in term of age, performance status, focal neurological signs, tumor resection, and histology. Male gender was more frequent in the 6-cycle group than the 12-cycle group. **Table 1** reveals the demographic and clinical characteristics of the two groups.

**Table 1:** The characteristics of the patients at baseline.

Characteristics	Entire group: 95 patients		
	TMZ 6-cycle, 46 patients n (%)	TMZ 12-cycle, 49 patients n (%)	P value
**Male Gender**	37(80.4)	28 (57.1)	0.015
**Age > 45**	22 (47.8)	25 (51)	0.75
**Karnofsky** **Performance Status ≥ 80%**	32 (69.5)	31 (63.2)	0.43
**Focal neurological deficits**	16 (34.8)	18 (36.7)	0.83
**Tumor resection:** Gross total Subtotal Biopsy only	8 (17.4) 28 (60.9) 10 (21.7)	9 (18.4) 24 (49) 16 (32.7)	0.43
**Histology:** Glioblastoma Anaplastic astrocytoma	37 (80.4) 9 (19.6)	40 (81.6) 9 (18.4)	0.82

YMZ: temozolomide.

### 3.2 The delivery of treatment

The median number of chemotherapy courses in the 6-cycle and 12-cycle groups were 5 (range: 1 to 6) and 10 (range: 2-12) respectively. Overall, 77/95 patients (81%) completed 6 courses of chemotherapy without progression. **Table 2** shows events that caused adjuvant treatment cessation (progression and/or death) before each cycle for both groups.

**Table 2:** The discontinuation rate of temozolomide per courses during the adjuvant treatment.

	6-cycle group n (%)	Cause of discontinuation	12-cycle group n (%)	Cause of discontinuation
Cycle 1	1 (2.2)	Death	0	–
Cycle 2	1 (2.2)	Death	2 (4.1)	Death (1)/Disease progression (1)
Cycle 3	0	–	5 (10.2)	Death (4)/Disease progression (1)
Cycle 4	0	–	2 (4.1)	Death (1)/Disease progression (1)
Cycle 5	2 (4.3)	Death	2 (4.1)	Death (2)
Cycle 6	0	–	3 (6.1)	Death (1)/Disease progression (2)
Cycle 7	–	–	2 (4.1)	Death (2)
Cycle 8	–	–	3 (6.1)	Death (1)/Disease progression (2)
Cycle 9	–	–	2 (4.1)	Disease progression (2)
Cycle 10	–	–	0	–
Cycle 11	–	–	1 (2)	Disease progression
Cycle 12	–	–	0	–

### 3.3 Survival and progression

**Figure 2:**
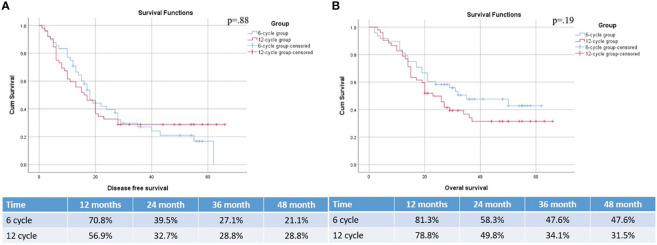
PFS **(A)** and OS **(B)** of patients with HGG based on the treatment groups.

With a median follow-up of 26 months, the 12-, 24-, 36-, and 48- month OS rates in 6-cycle and 12-cycle groups were 81.3% vs 78.8%, 58.3% vs 49.8%, 47.6% vs 34.1%, and 47.6% vs 31.5%, respectively (p=.19). Median OS of 6 cycles and 12 cycles groups were 35 months (95% confidence interval (CI), 11.0 to 58.9) and 23 months (95%CI, 16.9 to 29.0). The 12-, 24-, 36-, and 48- month PFS rates in 6 cycles and 12 cycles groups were 70.8% vs 56.9%, 39.5% and 32.7%, 27.1% vs 28.8%, and 21.1% vs 28.8%, respectively (p=.88). Median PFS of 6 cycles and 12 cycles groups were 18 months (95% CI, 14.8 to 21.1) and 16 (95% CI, 11.0 to 20.9) months (**Figure 2**).

Univariant regression analysis showed that male gender (hazard ratio (HR) 2.5, p=.03), age below 45-year-old (HR.39, p=.01), performance status (HR.95, p=.003), and histology of glioblastoma multiform (HR 4.5, p=.03) are the main predictors of survival. However, in multivariant regression analysis, performance status remained a significant predictor of survival survival (HR.9, p=.02) (**Table 3**).

**Table 3:** regression analysis of contributing factors to overall survival of patients with high-grade glioma.

	Univariant analysis			Multivariant analysis		
	HR	95% CI	P value	HR	95% CI	P value
Group of study (6-cycle)	.989	.48-2	.97			
Gender (male)	2.5	.96-6.6	.03	2.5	.9-6.5	.06
Age (<45 years old)	.39	.18-.84	.01	.5	.2-1.1	.08
Performance status (%)	.95	.91-.98	.003	.9	.92-.99	.02
Preoperative tumor size (cm)	1	.88-1.2	.658			
Peritumoral edema (negative)	.39	.13-1.1	.08			
Midline shift (negative)	.5	.21-1.1	.1			
Histology (GBM)	4.8	1.1-20.4	.03	3.5	.8-15.3	.08
Type of surgery (biopsy and STR)	1.5	.5-4.4	.4			
CTV (cm3)	1	.9-1	.5			

### 3.4 Safety

The most serious toxicity was grade 3 neutropenia, which was observed in 2 patients of the 6-cycles group and grades 3 and 4 thrombocytopenia, which were observed in 2 and 1 patients respectively in the 6-cycles group. Other toxicities were illustrated in **Table 4**. As shown in the table, only mild adverse events were relatively more frequent in 12-cycle group.

**Table 4:** Treatment toxicity in patients treated with 6 or 12 cycles of temozolomide.

Adverse event	6-cycle group 45 patients N (%)				12-Cycle group 49 patients N (%)			
	Grade 1	Grade 2	Grade 3	Grade 4	Grade 1	Grade 2	Grade 3	Grade 4
**Neutropenia**	39 (84.8)	5 (10.9)	2 (4.3)	0	46 (95.8)	2 (4.2)	0	0
**Thrombocytopenia**	39 (84.8)	4 (8.7)	2 (4.3)	1	44 (91.7)	4 (8.3)	0	0
**Anemia**	44 (95.7)	2 (4.3)	0	0	47 (97.9)	1 (2.1)	0	0
**Nausea/vomiting**	36 (87.2)	3 (6.6)	0	0	45 (95.7)	2 (4.2)	0	0

## 4 Discussion

This randomized study in glioblastoma patients who had undergone maximum safe surgical resection and who had completed adjuvant chemoradiation, did not show benefit from extended adjuvant temozolomide compared to the standard adjuvant course of temozolomide in terms of overall survival or progression free survival. We designed the randomization on an intent-to-treat basis to avoid selection bias. Therefore, patients who failed to complete adjuvant chemotherapy for any reason were not excluded from analysis.

In a retrospective study, Seiz et al. evaluated a group of 114 newly diagnosed glioblastoma patients treated by maximum safe surgical resection, temozolomide based chemo-irradiation and adjuvant temozolomide (TMZ). The adjuvant chemotherapy continued until tumor progression or appearance of intolerance. They found a significant correlation between median time to progression (TTP) as well as overall survival (OS) and the number of chemotherapy cycles (17). However, given the retrospective nature of the study, a selection bias might be a limiting factor, as long survivors had more chance to receive more extended cycles of chemotherapy. In another retrospective cohort study by Skardelly et al. (14), 107 patients with glioblastoma were divided into three groups of receiving less than 6 cycles (Group A), exactly 6 cycles (group B), and more than 6 cycles (group C). The decision to continue or stop adjuvant temozolomide was based on physician’s discretion. The 12.7 month overall in group A was significantly lower than group B (25.2 months) and C (28.6 months). Patients in group C were younger than group B (age less than 50, 57.7% vs. 18.7%). Multivariate Cox regression did not prove an overall survival advantage for group C against group B. At the time of first progression, the response rate to TMZ/lomustine rechallenge was higher in group B than group C (47% versus 13%). The lower survival rate in group A can be attributed to unresponsive tumors causing early progression and/or unfavorable individual prognostic factors.

There are retrospective studies that analyzed glioblastoma patients who remained progression free at the end of 6 cycles of adjuvant TMZ therapy. In an analysis of a German Glioma Network cohort, Gramatzki et al. identified 142 patients who were progression free at 6 cycles of adjuvant TMZ, among whom 61 continued the treatment to at least 7 maintenance cycles (median 11, range 7-20). After adjusting for age, extent of resection, Karnofsky performance status, and presence of residual tumor and O^6^-methylguanine DNA methyltransferase *(MGMT)* promoter methylation status, no significant difference in OS (HR = 1.6, 95% CI: 0.8-3.3; P = .22) and PFS (HR = 0.8, 95% CI: 0.4-1.6; P = .56) was detected between two groups (18). Roldán Urgoiti et al. (19) identified a cohort of 273 glioblastoma patients by Alberta Cancer Registry among whom 52 (19%) underwent surgery, chemoradiation and were progression free at 6 cycles of adjuvant therapy. They found that patients who received more than 6 cycles (median 11, range 7-13) had significantly more favorable median survival than those receiving 6 cycle according to the Stupp protocol (3) (24.6 versus 16.5 months respectively, p=0.031). According to the authors, their institution amended their policy to allow physician to extend adjuvant chemotherapy up to 12 cycles provided patients had no progression and minimal toxicity. Therefore, physician’s discretion played a role in selecting patients for extended treatment probably those with more favorable general condition at the end of 6 cycles.

In several studies, patients who completed 6 cycles of adjuvant TMZ without progression were divided into two groups of continued versus no further treatment and prospectively analyzed. Blumenthal et al. performed a retrospective meta-analysis of 4 prospective clinical trials for newly diagnosed patients with glioblastoma who were progression free at least 28 days after cycle 6 of adjuvant temozolomide (20). Patients receiving 6 cycles were compared with those who continued treatment beyond 6 cycles. The decision to continue treatment was based on physician’s discretion. Among 624 patients eligible for analysis, 291 continued the treatment up to progression or 12 cycles. Patients who treated more than 6 cycles had significantly more favorable progression-free survival (hazard ratio 0.8, [0.65-0.98] p=0.03), but no significant difference in overall survival was detected (hazard ratio 0.92 [0.71-1.19], p=0.52). The physicians’ discretion for continuing treatment can cause a selection bias in favor of the group receiving beyond 6 cycles. In a multicentric randomized trial in Spain by Balana et al. (21), Patients who were progression free at cycle 6 of adjuvant therapy were randomly assigned to stop group (79 patients) and extended group (80 patients). The chemotherapy in the extended treatment group continued until 12 courses or progression. Extended treatment was not associated with a significant benefit in terms of 6-months survival rate (61.3% vs 55.7%). Hematological toxicity, albeit being mild, was more frequent in the extended arm.

Some studies randomized newly diagnosed patients at diagnosis or at the chemoradiation termination into two groups of 6-cycle and more than 6 cycles of adjuvant TMZ therapy with intention to treat (ITT) basis. In a study in India, Bhandari et al. randomized 40 patients after chemo-irradiation into 6-cycle and 12-cycle groups. The median number of adjuvant chemotherapy in 6-cycle and 12-cycle groups was 6 (range, 3-6) and 12 (3-12) respectively. Patients in the 12-cycle group showed more favorable PFS (12.6 vs 16.8 months, P=0.069) and OS (15.4 vs 23.8 months, P=0.044). However, in a meta-analysis, Gupta et al. (22) considered four randomized clinical trials that recruited newly diagnosed patients with glioblastoma following concurrent chemoradiation and found different results. 358 eligible patients were randomly assigned to 6 cycles or > 6 cycles. The two groups (> 6 cycles vs 6 cycles) had no significant difference in terms of risk of progression (HR=0.82, 95% CI:0.61-1.1 P=0.18) or death (HR=0.87, 95% CI: 0.6-1.27, p=0.12). Overall, the authors concluded that their data did not suggest benefits from extending treatment, especially when considering possible increased toxicity to patients, and enhanced cost for the health system. In another meta-analysis by Attarian et al. (23) of four randomized studies consisting of 882 glioblastoma patients in total, no significant difference in PFS [(12.0 months (95% CI 9.0 to 15.0) vs. 10.0 months (95% CI 7.0 to 12.0), P = 0.270] and OS [23.0 months (95% CI 19.0 to 27.0) vs 24.0 months (95% CI 20.0 to 28.0), P = 0.73] was found between patients assigned to 6-cycle or extended adjuvant treatment.

Previous studies have demonstrated that the methylation of the *MGMT* gene promoter is a significant predictor of survival in patients receiving concurrent and adjuvant temozolomide therapy (3, 24). The result of the meta-analytic study by Blumenthal et al. (20) suggested that patients with methylated *MGMT* promoter status may particularly benefit from extended treatment in terms of progression-free survival (HR 0.65 [0.50–0.85], *P <.*01); however, overall survival was not affected by *MGMT* promoter methylation. There is no randomized trial to assess if extended treatment is particularly beneficial in patients whose tumors exhibit methylated MGMT promoters. The lack of information regarding methylation status of tumors is one of the limitations of our study. However, there is no recommendation for selecting patients for the current standard treatment based on methylation status. Pseudo-progression, which occurs in 20% to 30% of patients with glioblastoma following the Stupp regimen, may present a clinical conundrum (25) and affect progression-free survival (PFS) analysis. However, randomization of patients mitigates the effect of pseudo-progression on comparing groups analysis. Moreover, overall survival (OS) is a more robust endpoint.

Main limitation of present study is not assessing IDH mutation and MGMT status. In fact, a median OS of 35 months in patients undergoing to 6 cycles of TMZ might be influenced by IDH mutation since none of the patients are reported to undergo to tumor treating fields; while a median OS of 23 months may be related with a high prevalence of MGMT methylated patients, for example. About MGMT status, it is worth mentioning that patients with MGMT hypermethylation might benefit from extended TMZ schedule; but in this study there are no stratifications about MGMT status. This is important since mixing up all HHG patients receiving TMZ may led to an erroneous interpretation of the results. In fact, it may be found that MGMT hypermethylated patients may benefit from an extended TMZ schedule (or maybe not). The reason behind it is that the study was conducted between 2018 and 2020 when HGG patients were evaluated based on WHO 2016 Classification of CNS Tumours Diffuse astrocytic and oligodendroglial tumours and the has been reported two years later to observe enough events. Therefore, measurement of genes/molecular profile alterations were not mandatory. On the other hand, number of patients who underwent gross total resection was substantially lower from other trials and it may limit the extrapolation of our study.

Overall, consistent with the results of our study, most high-quality randomized trials and meta-analytic studies do not support a significant benefit for the extended adjuvant therapy, especially when considering higher toxicity (albeit mild) and economic burden on the patients, society and health system. The survival gains from the extended therapy, if any, has been small, which significantly reduces the cost-benefit of the continued treatment. Moreover, the extended treatment may reduce response to salvage treatment, which may explain the lack of OS benefit for extended therapy despite having a small PFS superiority in some studies. However, a subgroup of patients with special molecular characteristics (26–28) might still benefit significantly from the extended treatment and or novel treatment modalities; this hypothesis warrants further investigation.

## Data availability statement

The raw data supporting the conclusions of this article will be made available by the authors, without undue reservation.

## Ethics statement

The studies involving humans were approved by the Ethics Committee of Mashhad University of Medical Sciences. The studies were conducted in accordance with the local legislation and institutional requirements. The participants provided their written informed consent to participate in this study.

## Author contributions

KA: Data curation, Funding acquisition, Investigation, Methodology, Project administration, Supervision, Validation, Writing – original draft, Writing – review & editing. MT: Conceptualization, Formal analysis, Funding acquisition, Investigation, Methodology, Project administration, Supervision, Validation, Visualization, Writing – original draft, Writing – review & editing. MS: Data curation, Investigation, Methodology, Writing – original draft, Writing – review & editing. SJ: Conceptualization, Investigation, Methodology, Writing – original draft, Writing – review & editing. HS: Investigation, Methodology, Writing – original draft, Writing – review & editing. JW: Supervision, Validation, Writing – original draft, Writing – review & editing.
